# A Rare Case of Spinal Cord Injury Following Thoracic Radiofrequency Ablation

**DOI:** 10.7759/cureus.15380

**Published:** 2021-06-01

**Authors:** Nicholas K Donohue, Christopher White

**Affiliations:** 1 Physical Medicine and Rehabilitation, Medical College of Wisconsin, Wauwatosa, USA

**Keywords:** spinal cord injury, mid back pain, radiofrequency ablation (rfa), physical medicine and rehabilitation, painful neuropathy, neurogenic bowel/bladder

## Abstract

Medial branch radiofrequency ablation (RFA) has become a common treatment for facet-related back pain. While this procedure is often performed in the lumbar and cervical spinal segments, it can also be applied to the thoracic spine. Complications of spinal RFA at any level are scarce in the literature and are often mild.

The patient is a 37-year-old male with a family history of multiple sclerosis in his siblings who underwent thoracic RFA at the T2-T5 nerve root levels. Within one week of the procedure, the patient experienced paresthesias below the nipple line and progressive lower-extremity weakness. He was also found to exhibit urinary retention on presentation to our facility one month later. MRI showed focal cord short T1 inversion recovery (STIR) signal abnormality at the T3-T4 level, favored to represent myelomalacia. An extensive laboratory and imaging workup was otherwise unrevealing. The patient was treated with neuromodulators and a short course of inpatient rehabilitation. One year later, he used knee-ankle-foot orthoses for ambulating short distances and a manual wheelchair for longer distances, and he no longer required intermittent catheterization for bladder management.

This case presents a rare and unusual timeline of symptom evolution, laboratory findings, and imaging results that do not unveil a clear pathophysiological mechanism, which led to the patient’s spinal cord injury. The clinical level of injury based on the patient’s symptoms and location of myelomalacia on MRI, however, strongly support a causative contribution by the thoracic RFA procedure.

## Introduction

Radiofrequency ablation (RFA) of the medial branch of the spinal nerve dorsal ramus has become a common treatment for facet-related back pain [1,2]. While this procedure is often performed in the lumbar and cervical spinal segments, it can also be applied to the thoracic spine. Complications of spinal RFA at any level are scarce in the literature and are often mild. The most commonly reported complications of cervical RFA are postprocedural pain, cutaneous numbness, dizziness, and dysesthesias [3]. To our knowledge, there are no published reports of spinal cord injury (SCI) following spinal RFA.

## Case presentation

The patient is a 37-year-old male with a past medical history of attention-deficit disorder and asthma, and a family history of multiple sclerosis (MS) in his twin brother and sister. The patient had established care with an outside pain medicine provider six months prior to the injury. At that time, he reported a 10-year history of intermittent thoracic spine pain following a car accident that was associated with numbness and tingling across his midback. He had recently undergone magnetic resonance imaging (MRI) of his thoracic spine, which showed mild degenerative changes including a small central disc bulge at the T3-T4 level with no significant central canal or foraminal narrowing. He was started on scheduled tramadol and gabapentin and underwent interlaminar epidural steroid injections at the T3-T4, T4-T5, and T8-T9 levels over the subsequent months with minimal pain or functional improvement. At his four-month follow-up, his radicular symptoms had significantly improved, but he continued to complain of severe axial pain in his thoracic spine. He subsequently underwent bilateral medial branch blocks at the T2-T5 nerve levels since the worst of his pain was localized to the bilateral T3-T4 and T4-T5 facets. He received more than 80% temporary pain relief with the diagnostic blocks and subsequently underwent RFA to the right side, followed two weeks later on the left side.

Two days following his second thoracic RFA procedure, the patient presented to an outside emergency department (ED) complaining of severe, axial thoracic spine pain in the area of his recent procedure. He specifically denied any radiation, lower-extremity weakness, numbness, or tingling. He underwent a thoracic CT with no abnormalities seen and was discharged home with a steroid taper pack. He presented again to the ED seven days after the second RFA procedure, now complaining of diffuse paresthesias from his upper abdomen to his feet and subjective weakness in his bilateral lower extremities. MRI thoracic spine was performed and it only showed soft tissue hyperintensity signals on T2-weighted imaging in the paraspinal muscles at the level of recent RFAs (left worse than right). The patient was discharged with the recommendation to follow up with his pain provider. He did see the outside pain provider, who also ordered an MRI thoracic spine 13 days post-RFA, which again showed similar thoracic paraspinal edema and no cord signal change. He was advised to continue a steroid taper and follow up in three weeks.

**Figure 1:**
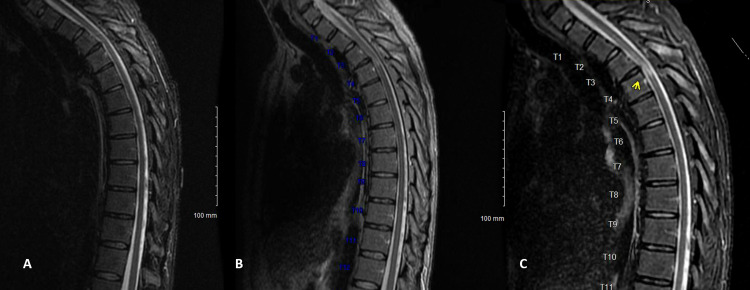
Serial Throracic MRI Sequences Short T1 inversion recovery sequencing sagittal MRI views of the patient’s thoracic spine at A) one week, B) one month, and C) two months after the thoracic radiofrequency ablation procedure. Cord signal changes were not present at one week but were seen in the one-month study and had slightly increased at two months (arrow indicating location of cord signal changes).

The patient presented to the ED of our institution four weeks following his left RFA procedure with similar complaints of paresthesias below his chest and worsening bilateral lower-extremity weakness. He did also endorse new complaints of constipation and difficulty initiating micturition. Physical examination was notable for 1-2/5 strength diffusely in bilateral lower extremities, 3+ patellar reflexes, sustained bilateral ankle clonus, positive Babinski sign bilaterally, and decreased light touch and pinprick sensation in bilateral lower extremities. A bladder scan also showed 1 L urine that required straight catheterization. He was admitted to the neurology service and underwent another MRI thoracic spine. This MRI did show focal cord short T1 inversion recovery (STIR) signal abnormality at the T3-T4 level with no enhancement or restricted diffusion, favored to represent myelomalacia (Figure 1). There was no restricted diffusion to suggest infarct, and no cord expansion or enhancement to suggest acute myelitis. Magnetic resonance angiography was performed on day 3 of admission to assess for possible dural arteriovenous (AV) fistula, which was negative. A lumbar puncture was performed on day 5 of admission, and the extensive cerebrospinal fluid (CSF) testing was largely unrevealing. Specifically, all bacterial, viral, and fungal testing were negative, there were no oligoclonal bands or elevated immunoglobulin G (IgG) index, and there was no albuminocytologic dissociation. He then underwent a spinal angiogram with interventional radiology on day 8, which was negative for subdural AV fistula. The patient had stable symptoms and was admitted to our inpatient rehabilitation service on day 10 of his acute admission. After four days, he got discharged home prematurely to attend a family event. At that time he primarily required a manual wheelchair for mobility and was on an extensive medication regimen to address deficits involving bowel and bladder management, neuropathic pain, and lower-extremity spasticity. He was followed closely by a fellowship-trained SCI physiatrist over the next year. One year following the injury, his physical examination revealed decreased sensation to light touch and pinprick from T4-S5 dermatomes, 4/5 strength diffusely in bilateral lower extremities, 3+ patellar and Achilles reflexes, and bilateral ankle clonus. He used knee-ankle-foot orthoses for ambulating short distances and a manual wheelchair for longer distances, and he no longer required ISC for bladder management.

## Discussion

The above case is a rare instance of SCI following thoracic medial branch RFA. To our knowledge, there are no formal reports of such a case in any literature.

Complications of medial branch RFA have been sparsely reported in the past. Engel et al. performed a systematic review of the effectiveness and risks of cervical medial branch RFA [3]. Their extensive literature search yielded only four research articles that reported complications of the procedure [3]. In descending order of incidence, they reported the most common complications as postprocedural pain, cutaneous numbness, dizziness, and dysesthesias, all of which occurred in 30%-98% of procedures [3]. The rare complications that occurred in 1%-3% of cases included postprocedural infection, neuritis, dermoid cyst, and Köbner’s phenomenon, which is the development of a skin lesion following trauma [3]. The authors then reference a review paper from 2008 by Bogduk et al. that narrates two cases of SCI following cervical RFA [4]. These cases were only known to the authors “by way of medicolegal proceedings” [4]. The first case describes a patient who developed Brown-Séquard syndrome following C3-C4 RFA. The intraoperative films showed misplaced electrodes that had passed between the cervical laminae and entered the spinal cord. Additionally, this procedure was performed under general anesthesia, so the patient could not report any pain or abnormal sensation during the procedure itself. The second case was of a patient who had MRI findings of severe central cord edema following a third occipital nerve RFA [4]. An anterior-posterior X-ray from the procedure that was available to the authors showed that the electrode tip had been advanced into the intervertebral foramen. The authors concluded that all available imaging were suggestive of coagulation of a reinforcing radicular artery that resulted in spinal cord infarction [4]. Unfortunately, the patient’s symptoms and timing of the postprocedural MRI were not reported in this review [4].

There are several aspects of this case that do not fit a clear mechanism of injury, which led to the patient’s SCI. First, there is the timeline of the patient’s symptoms. The patient did not report any new symptoms until two days after the RFA, and even then his only symptom was left-sided axial thoracic back pain. He did not report new neurological deficits of paresthesias and lower-extremity weakness until seven days after the procedure. He did, of note, receive a steroid taper pack at his initial ED presentation, and this may have impacted the timeline of his symptoms to some degree. The chronology of symptoms from thermal ablation of the spinal cord itself or related vasculature is not known. The progression of symptoms following traumatic SCI is well documented and generally immediate, beginning with spinal shock [5]. Other forms of SCI (e.g., as transverse myelitis, MS, or Guillain-Barré Syndrome (GBS)) tend to have correlating imaging and laboratory findings that support the history and physical examination findings, as discussed below.

The second aspect of this case that may not fit a clear mechanism of injury to the spinal cord is the timing of imaging findings. As stated above, the patient underwent two thoracic MRIs within seven days of the RFA that showed no cord signal changes. It was not until he presented to our institution four weeks post procedure that a new finding of increased STIR signal, suggestive of myelomalacia, was seen at the level of the RFA. There was also no sign of infarction (acute or chronic) on diffusion-weighted imaging, and the location of the lesion does not match an arterial territory, so arterial compromise during the RFA is unlikely. Upon review of the literature, however, spinal cord enhancement and expansion may be less common in acute transverse myelitis than previously thought. In one series of 170 patients with idiopathic transverse myelitis, T2-weighted imaging showed a signal abnormality in 44% of cervical injuries and only 37% of thoracic injuries [6]. Additionally, Scotti and Gerevini performed a literature review on MRI findings in acute transverse myelitis and concluded that positive findings occur in only about 40% of cases [7]. Thus, in theory, the patient in this case may have experienced an episode of transverse myelitis correlating both to the procedure and his symptom onset that only appeared as tissue necrosis or myelomalacia four weeks later. 

The third confounding part of this case that does not lead to a clear mechanism of injury is the unrevealing CSF labs obtained during the patient’s admission. Oligoclonal bands have been reported in 85%-95% of MS cases, and an elevated IgG index is present in up to 75% of the time [8,9]. CSF abnormalities in acute transverse myelitis include pleocytosis and increased protein levels [10]. A retrospective review of 457 transverse myelitis cases found that only 57% of patients that met criteria for inflammatory myelopathy actually had abnormal CSF findings [11]. In patients with GBS, lumbar puncture often reveals an elevated CSF protein with a normal CSF white blood cell count [12]. This finding, known as albuminocytologic dissociation, is present in 50%-66% of patients with GBS in the first week after the onset of symptoms and ≥75% of patients in the third week [12,13]. Therefore, a normal CSF analysis would make MS or GBS far less likely for the patient presented in this case. An episode of transverse myelitis four weeks prior, however, would be within the realm of possibility.

## Conclusions

This case represents a rare occurrence of SCI following thoracic RFA in the context of a patient with a strong family, but no personal, history of MS. The timing and location of the injury strongly support some causative relation of the SCI to the RFA procedure, but an extensive imaging and laboratory workup does not suggest a clear mechanism of injury.

## References

[1] Barreras P, Fitzgerald KC, Mealy MA (2018). Clinical biomarkers differentiate myelitis from vascular and other causes of myelopathy. Neurology.

[2] Ditunno JF, Little JW, Tessler A, Burns AS (2004). Spinal shock revisited: a four-phase model. Spinal Cord.

[3] Dobson R, Ramagopalan S, Davis A, Giovannoni G (2013). Cerebrospinal fluid oligoclonal bands in multiple sclerosis and clinically isolated syndromes: a meta-analysis of prevalence, prognosis and effect of latitude. J Neurol Neurosurg Psychiatry.

[4] Bogduk N, Dreyfuss P, Baker R, Yin W, Landers M, Hammer M, Aprill C (2008). Complications of spinal diagnostic and treatment procedures. Pain Medicine.

[5] Engel A, Rappard G, King W, Kennedy DJ (2016). The effectiveness and risks of fluoroscopically-guided cervical medial branch thermal radiofrequency neurotomy: a systematic review with comprehensive analysis of the published data. Pain Med.

[6] Falco FJ, Manchikanti L, Datta S (2012). Systematic review of the therapeutic effectiveness of cervical facet joint interventions: an update. Pain Physician.

[7] Giesser BS (2011). Diagnosis of multiple sclerosis. Neurol Clin.

[8] Krishnan C, Kaplin A, Calabresi P, Kerr D (2004). Clinical characteristics and prognostic factors in 170 patients with idiopathic transverse myelitis. Neurology.

[9] Leggett LE, Soril LJ, Lorenzetti DL, Noseworthy T, Steadman R, Tiwana S, Clement F (2014). Radiofrequency ablation for chronic low back pain: a systematic review of randomized controlled trials. Pain Res Manag.

[10] Nishimoto Y, Odaka M, Hirata K, Yuki N (2004). Usefulness of anti-GQ1b IgG antibody testing in Fisher syndrome compared with cerebrospinal fluid examination. J Neuroimmunol.

[11] Scotti G, Gerevini S (2001). Diagnosis and differential diagnosis of acute transverse myelopathy. The role of neuroradiological investigations and review of the literature. Neurol Sci.

[12] Sellner J, Lüthi N, Schüpbach WM (2009). Diagnostic workup of patients with acute transverse myelitis: spectrum of clinical presentation, neuroimaging and laboratory findings. Spinal Cord.

[13] Yuki N, Hartung HP (2012). Guillain-Barré syndrome. N Engl J Med.

